# Comparing hair-morphology and molecular methods to identify fecal samples from Neotropical felids

**DOI:** 10.1371/journal.pone.0184073

**Published:** 2017-09-07

**Authors:** Carlos C. Alberts, Bruno H. Saranholi, Fernando Frei, Pedro M. Galetti

**Affiliations:** 1 LEvEtho (Laboratory of Evolution and Ethology), Faculdade de Ciências e Letras de Assis, Universidade Estadual Paulista, Assis, State of São Paulo, Brazil; 2 LabBMC (Laboratório de Biodiversidade Molecular e Conservação), Departamento de Genética e Evolução, Universidade Federal de São Carlos, São Carlos, State of São Paulo, Brazil; 3 LEA (Laboratório de Estatística Aplicada), Faculdade de Ciências e Letras de Assis, Universidade Estadual Paulista, Assis, State of São Paulo, Brazil; Agency for Science Technology and Research, SINGAPORE

## Abstract

To avoid certain problems encountered with more-traditional and invasive methods in behavioral-ecology studies of mammalian predators, such as felids, molecular approaches have been employed to identify feces found in the field. However, this method requires a complete molecular biology laboratory, and usually also requires very fresh fecal samples to avoid DNA degradation. Both conditions are normally absent in the field. To address these difficulties, identification based on morphological characters (length, color, banding, scales and medullar patterns) of hairs found in feces could be employed as an alternative. In this study we constructed a morphological identification key for guard hairs of eight Neotropical felids (jaguar, oncilla, Geoffroy’s cat, margay, ocelot, Pampas cat, puma and jaguarundi) and compared its efficiency to that of a molecular identification method, using the ATP6 region as a marker. For this molecular approach, we simulated some field conditions by postponing sample-conservation procedures. A blind test of the identification key obtained a nearly 70% overall success rate, which we considered equivalent to or better than the results of some molecular methods (probably due to DNA degradation) found in other studies. The jaguar, puma and jaguarundi could be unequivocally discriminated from any other Neotropical felid. On a scale ranging from inadequate to excellent, the key proved poor only for the margay, with only 30% of its hairs successfully identified using this key; and have intermediate success rates for the remaining species, the oncilla, Geoffroy’s cat, ocelot and Pampas cat, were intermediate. Complementary information about the known distributions of felid populations may be necessary to substantially improve the results obtained with the key. Our own molecular results were even better, since all blind-tested samples were correctly identified. Part of these identifications were made from samples kept in suboptimal conditions, with some samples remaining outdoors for up to seven days, simulating conditions in the field. It appears that both methods can be used, depending on the available laboratory facilities and on the expected results.

## Introduction

Traditional behavioral-ecology studies of mammalian predators require often expensive, difficult and invasive methods such as radiotelemetry (or satellite position systems), where the animal must be captured, sedated, attached to uncomfortable equipment, and then followed or its position tracked. More recently, less-invasive methods have been employed, such as camera traps and analysis of DNA from organic samples left by individuals [1–4].

Whereas traditional methods provide only limited ecological information on the target species or are not cost-effective, analysis of the DNA found in feces, hair bulbs, hair shafts [5] and other tissues of the animal make it possible to identify the species, identity and sex. This allows inferences about the degree of relatedness among individuals, the population size and the genetic inbreeding level, and the composition of their diets [6]. However, the species must first be identified. Species identification based on molecular data from feces is relatively simple and cost-effective, and this method can often indicate the exact species, using specific primers [3, 7]. In the case of Neotropical species, the most common molecular markers utilized for identification are from the cytochrome b region [2, 8] and, more recently, primers for a region of ATP6 have also been employed [3, 9]. This can be termed a DNA mini-barcoding approach, since it makes use of a short segment from mitochondrial DNA for species identification [3]. The often poor quality of DNA obtained from fecal samples (the DNA is degraded by environmental conditions in the field) necessitates the use of short sequences for molecular identification, and primers that amplify short sequences, generally fewer than 200 bp. Primers from the ATP6 region described in [3], which amplify a 126-bp segment, are suitable. These primers have been successfully used to identify felid species from fecal samples [10–15], even for species with feces that are similar in morphology and size and hence difficult to distinguish in the field, such as the ocelot and puma [2].

This technique requires both the facilities of a molecular biology laboratory, and, also fresh fecal samples to avoid DNA degradation [2, 9], which is not easy to achieve in field expeditions.

In order to inform decisions about ongoing *“in situ”* research, a desirable method would allow almost immediate identification of felids, without requiring full laboratory facilities. One such method is the identification of mammals based on the morphology of their guard hairs. Trichology, the identification of hairs, is an important methodological alternative in mammal surveys and dietary studies [16–19]. Nonetheless, trichology as a method of identification has certain drawbacks. The most important one, according to some investigators [20], is that species identification is not completely certain, particularly in the case of closely related species with morphologically similar hairs. However, the molecular method may also not be completely reliable, since usually DNA cannot be successfully amplified from all the samples [7]. In any case, some studies have used trichology to identify mammal species in the Neotropical region [20–24] even being available a reliable molecular methodology to identify them from fecal samples [25–31]. Part of the literature on hair morphology provides identification keys [32–35].

Felid species, as mammals of the Order Carnivora, are generally rare, exist in low densities, and show secretive behavior [36, 37]. Felids are strictly carnivorous and their feces contain large amounts of prey hairs and/or feathers. Furthermore, felids display a characteristic, copious grooming behavior [38, 39]. In the process of grooming, shed hairs are swallowed and later appear in the feces. Therefore, the identification of guard hairs found in these feces can be an important tool to reveal the species of both the prey and the predator itself, and can be applied in ecological studies.

In this study, we compared methods of morphological and molecular identification of Neotropical felid species through fecal samples, and the efficiency of these methods in identifying species. For this, 1) we constructed and tested the effectiveness of a morphological hair identification key, and 2) developed and tested a protocol for fecal DNA extraction and molecular identification of Neotropical felid species.

## Methods

Using hairs collected from museum specimens and from feces of captive individuals of eight species of Neotropical felids, we constructed a morphological identification key using color, banding pattern, size, scale and medullar patterns. Using two different sets of samples of feces from these same captive individuals, we blind-tested the effectiveness of the key and compared it to the effectiveness of a molecular protocol. To perform the blind tests, each sample was coded with a number for each species. The codes were revealed only after the samples were identified with both methods.

### Felid species

Of the ten extant Neotropical felid species, we chose not to include the Andean mountain cat *Leopardus jacobita* and the Kodkod *Leopardus guigna*. These two species are restricted to high altitudes (above 4,100 m) and/or to the southernmost part of the Neotropical Region [40, 41] We concentrated on the eight species most likely to be found in the Neotropical region, the ocelot *Leopardus pardalis*, the margay *Leopardus wiedii*, the oncilla *Leopardus tigrinus*, the Geoffroy’s cat *Leopardus geoffroyi*, the Pampas cat *Leopardus colocolo*, the puma *Puma concolor*, the jaguarundi *Puma yagouaroundi* and the jaguar *Panthera onca*.

Most of these species are listed in CITES and/or the IUCN Red List, and are locally or globally endangered or critically endangered (Table 1).

**Table 1:** Extinction risk for each species used in this study, according to CITES and IUCN.

Species	CITES Status	IUCN Red list Status
*Leopardus pardalis*	Threatened with extinction	Least concern
*Leopardus wiedii*	Threatened with extinction	Near threatened
*Leopardus tigrinus*	Threatened with extinction	Vulnerable
*Leopardus geoffroyi*	Threatened with extinction	Least concern
*Leopardus colocolo*	CITES Appendix II	Near threatened
*Puma concolor*	CITES Appendix II	Near threatened
*Puma yagouaroundi*	Threatened with extinction	Least concern
*Panthera onca*	Threatened with extinction	Near threatened

Concerning *L*. *tigrinus*, a recent study [42] proposed that this species has a cryptic counterpart, *Leopardus guttulus*, in the more humid part of its formerly presumed distribution in Brazil, the Atlantic Rain Forest in the south/southeast region of the country. Discrimination between these two species and populations was made possible through the use of molecular markers [42], and also by means of morphological and behavioral characters, in an earlier review [43]. Individuals of *L*. *tigrinus* from other Neotropical areas, such as Central America, also show important molecular differences from *L*. *guttulus* [44, 45]. However, due to the absence of *L*. *guttulus* sequences in the database and the uncertainty of the geographic origin of some of the individuals kept in zoo, we opted to use the broader and still most-often applied nomenclature, *L*. *tigrinus*.

### Sampling strategy

To construct the identification key and to verify its efficiency and that of our molecular approach, we used a number and diversity of samples that seemed to be appropriate to encompass interspecific morphological and molecular variation, and were also feasible. As can be seen in our results, this strategy appeared to be sufficient to allow the identification of Neotropical felids. The sampling strategy is detailed below in this and subsequent sections.

As our purpose was to evaluate the efficiency of both molecular and morphological methods to identify Neotropical felids, we first constructed a morphological identification key (MIK) based on appropriate characters. We used only guard hairs to construct the key, as they are coarser and thus probably more tolerant to the environment of the gastric tube. Guard hairs also show more morphological characters that can be used to identify species. There is some intra-individual variation in these characters; however, the interspecific variation is definitively greater than the intraspecific variation, allowing species identification [46–47]. We used two sources of hairs. The first set of hairs was obtained from voucher specimens of the Museu de Zoologia da Universidade de São Paulo (MZUSP, São Paulo, Brazil). For each species we collected hairs from one male and one female individual, with the exception of *L*. *geoffroyi*, which at the time had only one male individual identified in the museum’s collection. For each species, hairs were mounted on 30 glass slides (15 slides for each sex) to analyze the cuticular scales, and another 30 slides (also 15 for each sex) to study medullar patterns; each slide contained only one hair. To investigate hair color patterns, from each species we used different numbers of guard hairs: *L*. *pardalis* (30 hairs), *L*. *wiedii* (49), *L*. *tigrinus* (30), *L*. *geoffroyi* (30), *L*. *colocolo* (36), *P*. *concolor* (42), *P*. *yagouaroundi* (43) and *P*. *onca* (52). To measure hair length, from each species we used: *L*. *pardalis* (24 hairs), *L*. *wiedii* (19), *L*. *tigrinus* (25), *L*. *geoffroyi* (22), *L*. *colocolo* (60), *P*. *concolor* (25), *P*. *yagouaroundi* (29) and *P*. *onca* (17). For these two types of patterns, color and length, also half of the hairs came from males and half from females, with the exception of *L*. *geoffroyi*. The second source of hairs used to construct the identification key was known fecal samples, from the same species, collected by the staff of the São Paulo Zoo (Fundação Parque Zoológico de São Paulo). For each species, we employed 10 hairs found in fecal samples, to observe the above-mentioned morphological characters. Samples were collected according to the availability of feces in selected enclosures, where specimens were held during the collecting period. Each enclosure contained one to several adult individuals of one of the species used here, belonging to one or both sexes (Table 2).

**Table 2:** Samples of feces collected from captive felids.

Species	Males	Females
*L*. *pardalis*	1	2
*L*. *wiedii*	1	3
*L*. *tigrinus*	15	12
*L*. *geoffroyi*	6	4
*L*. *colocolo*	5	3
*P*. *concolor*	2	0
*P*. *yagouaroundi*	4	1
*P*. *onca*	2	1

Therefore, and because we didn’t investigate molecularly this fecal material, we couldn’t discriminate the sex or the number of the individuals that contributed the samples.

### Constructing the morphological identification key

To construct the hair identification key, we employed guard hairs from samples collected as described above.

The nomenclature used to describe the morphological features of hairs was based on several studies [24, 48, 49].

**Fig 1:**
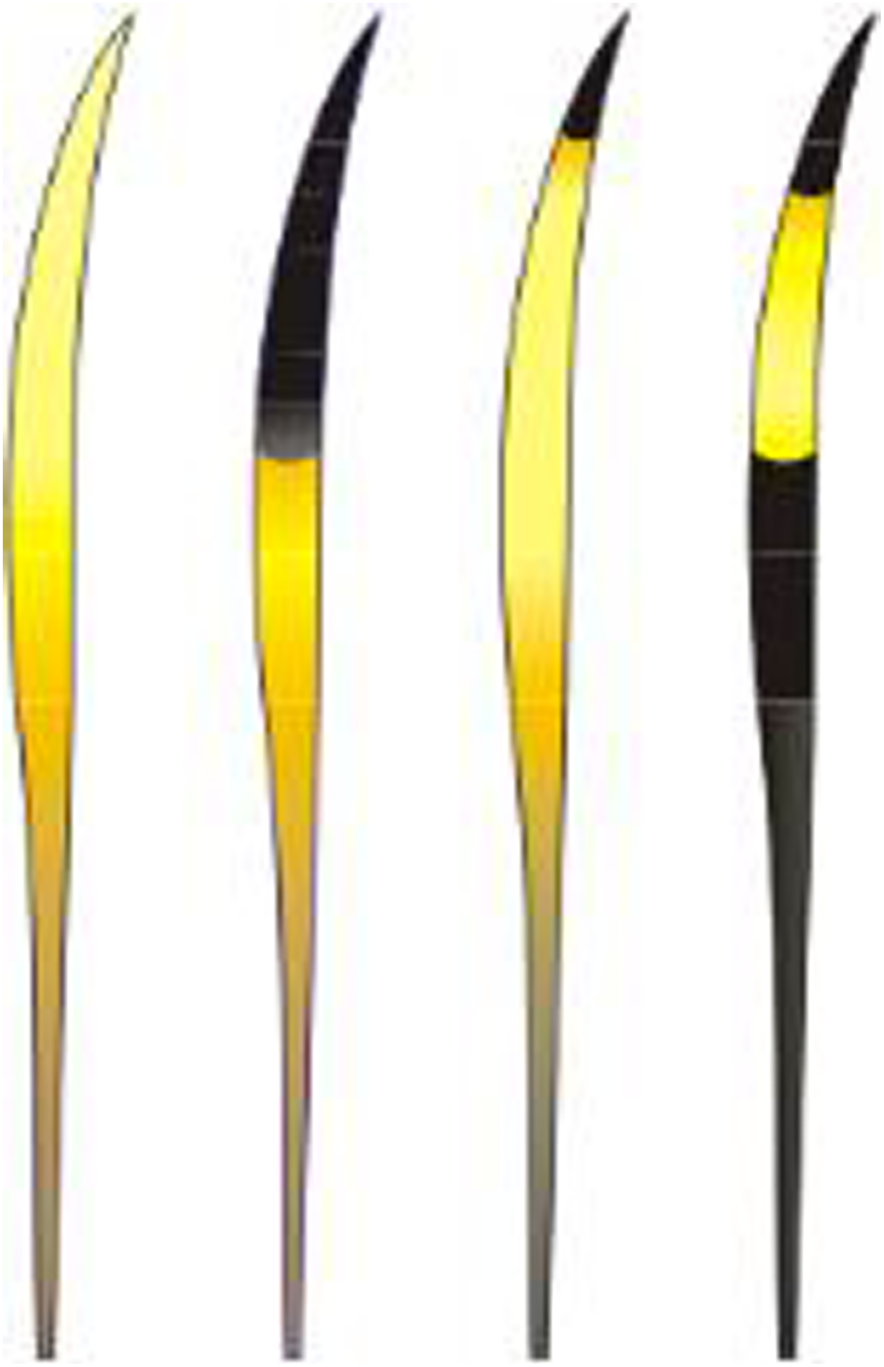
Example of coloration and banding patterns from guard hairs of Neotropical felids.

As mentioned above, we evaluated four morphological characteristics of guard hairs: banding color pattern, cuticle scales, medullar morphology and hair length. For the color and banding analyses (Fig 1), we observed hairs along their length from the proximal to the distal end, through a stereoscopic microscope with the aid of white light against a pale-blue background [50, 51]. Descriptions of color and banding refer only to the shield region of the guard hair. The color terminology used is as in the common sense (black, yellow, white, brown, etc.).

**Fig 2:**
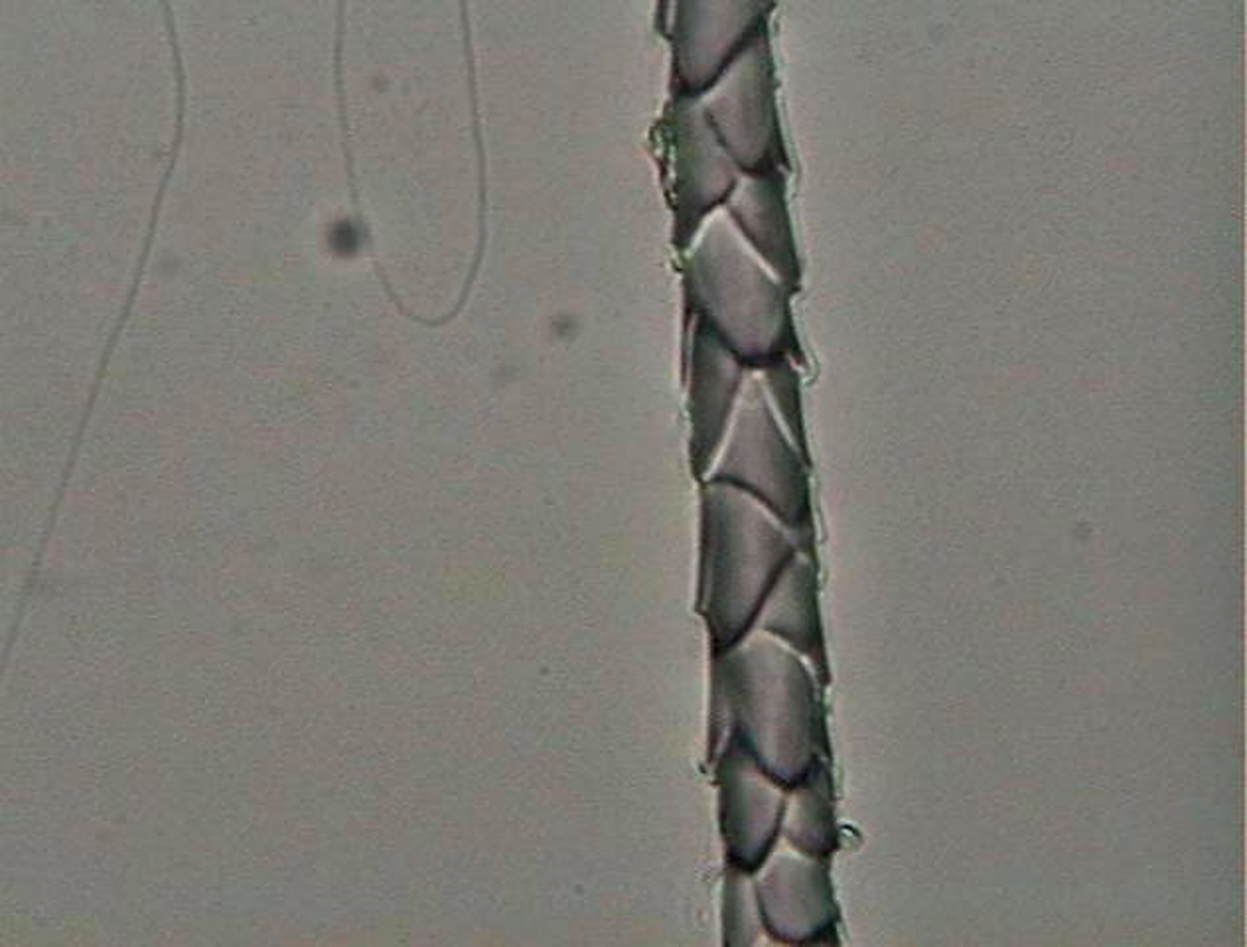
Example of preparation for scale-pattern analysis, in the shaft region, for a guard hair of *L*. *pardalis*.

The preparation for analyzing scale morphology was a traditional method [49]. It consisted of printing the hair pattern on a glass slide with the aid of clear nail enamel and an arm press. After the enamel dried, the hair was carefully removed and the resulting print could be analyzed under a light microscope (Fig 2).

To analyze the medullar pattern, the hair was bleached using oxygen peroxide cream, and then mounted in Canada balsam on a glass slide. It could then be viewed under light microscope [24].

To measure the length of guard hairs we used common steel rulers with a millimeter scale [24].

### Fecal DNA extraction and molecular identification

DNA was extracted following the recommendations of the QIAmp DNA Stool Mini Kit (Qiagen), a specific kit for extracting DNA from fecal samples. For this procedure, a small piece from the surface of the fecal sample is used for the DNA extraction. This methodology (ethanol preservation and Qiagen Kit) has been commonly used in other studies [25–28] for fecal sample preservation and DNA extraction. According to one study [25], which evaluated five methods of DNA preservation, ethanol was the best way to preserve feces for mitochondrial and nuclear DNA analyses. Another study [26] also found robust DNA extraction results from ethanol-preserved fecal samples, even at room temperature, and concluded that ethanol is a convenient medium for sample preservation, even in the field.

Although it would be desirable, DNA quantification and quality cannot be assessed after extraction when dealing with carnivores, because it is not possible to distinguish DNA from the predator (in this case the felid species) from the DNA of the prey species in its diet. For this reason, the success of the DNA extraction is evaluated only by amplifying the DNA in the PCR. In recent studies with fecal samples, DNA quantification and quality were also not measured after DNA extraction [3, 8, 17–20].

The species molecular validation was conducted by amplifying a sequence of 126 bp from mitochondrial DNA of the ATP6 gene region [3]. As mentioned above, this can be regarded as a mini-barcoding approach, since it uses a short segment from mitochondrial DNA for species identification.

The PCRs were performed in a Veriti 96-Well Thermal Cycler (Life Technologies) with the conditions: 3–5 μl of DNA, 1X PCR buffer, 100 μM of each dNTP, 8.0 pmol of each primer, 2.0 mM MgCl_2_, 1.0 U of Platinum^®^ DNA Polymerases (Invitrogen) in a final volume of 15 μl. Cycling conditions were as follows: initial denaturation at 94°C for 3 min, 94°C for 45 s, 60°C for 45 s (touchdown –1°C for 10 cycles), 72°C for 1 min 30 s, and 30 cycles at 94°C for 45 s, 50°C for 45 s, 72°C for 1 min 30 s, and a final extension at 72°C for 3 min. The PCR products were checked in 1% agarose gel with GelRed^™^ (Biotium) and purified using ExoSAP-IT (Affymetrix).

Amplification products were sequenced in an automated DNA sequencer (ABI3730XL, Applied Biosystems). The DNA sequences obtained were edited and matched with reference sequences (from samples of different species available from the tissue bank of our molecular laboratory) using the software GENEIOUS, v. 6.1.5 (Biomatters Ltd., Auckland, New Zealand). The identity match was verified by the neighbor-joining algorithm [30] with the Kimura 2-parameter model [31]. The support for clusters was assessed by bootstrap resampling (1000 resamplings). Sequences were considered to belong to the same species if they formed monophyletic groups with the reference sequences. This procedure has been used elsewhere for sample identification [2, 3, 8, 29]. Some sequences for the same species were previously deposited in GenBank by other investigators. Our own sequences have also been made available in GenBank (S1 Table)

### Testing efficiency of morphological and molecular methods

After the key was completed, we used two additional sets of samples to execute blind tests to check the MIK efficiency, and also to evaluate the efficacy of our molecular protocol as described above. To test the key, we requested the staff of the São Paulo Zoo to send additional fecal samples from the species of interest. They provided a total of 79, including 9 samples for one species and 10 for the others. These samples came from the same enclosures as the previous set of samples.

Feces were preserved in a 70% ethanol solution when samples were used to perform morphological identifications. Guard hairs were gathered with the aid of tweezers, and for each fecal sample, three slides were made for cuticle analysis and another three for medullar examination. The remaining guard hairs gathered from each sample were analyzed for color and banding pattern. Each fecal sample was identified with a code.

The set of samples that we used to blind-test the efficiency of our molecular method was also collected by the staff of the São Paulo Zoo, in the same zoo enclosures. It is therefore not possible to know whether the samples used to construct the MIK, those used to blind-test the key, and those used to blind-test the molecular protocol came from the same individuals.

In the case of blind-testing the molecular protocol, however, we used a procedure to also check DNA degradation in the fecal samples when they remain for a longer period in the field.

Normally, when possible, it is advisable to use the freshest possible samples to extract DNA from the cells of the integument of the digestive tract that lie on the surface of feces, to avoid deterioration of this material. It is also highly advisable to preserve the sample in a sterilizing storage medium, such as ethanol, and to freeze it immediately. However, in the field, fresh feces are rarely found. Most often, samples are days or even weeks old. Moreover, depending on the kind and duration of expeditions, it is usual that the collected samples are preserved in ethanol only a few hours after they are found and, further, are only sent to a laboratory a few days later, where they are finally to be frozen.

So, in our study, we attempted to simulate some of the conditions of the samples found in the field, and compared the efficiency of the procedures. The samples were collected from the enclosures of the species of interest, in the zoo, and preserved following three different protocols; collected fresh, preserved in 96% ethanol and immediately frozen; or preserved in ethanol immediately after they were freshly collected and put in the freezer only seven days later; or were collected, but left outdoors for seven days and then preserved in 96% ethanol and, only then, put in the freezer. It is important to note that while for molecular analyses, fecal samples must be stored in 96% ethanol to preserve DNA against degradation, for hair morphology analyses, samples can be stored in either 96% or 70% ethanol.

The samples (three of each kind) were then sent to our molecular laboratory (Laboratório de Biodiversidade Molecular e Conservação, Universdade Federal de São Carlos) for DNA extraction and analysis.

The tests of efficiency for both methods were blind, and the decoding for each sample’s code resting in sealed mailing envelopes, which were opened only when all samples had been identified. We categorized the efficacy of both methods through the division by percentage quintile of correct identification, using five categories: 0–19% (inadequate), 20–39% (poor), 40–59% (moderate), 60–79% (good), and 80–100% (excellent).

## Results

Guard hairs from the species analyzed showed great variation in the cuticle scale pattern along the length of each individual hair. Therefore, for practical purposes and for a more precise description, we considered three regions: shield; transitional region between shield and shaft; and shaft.

**Fig 3:**
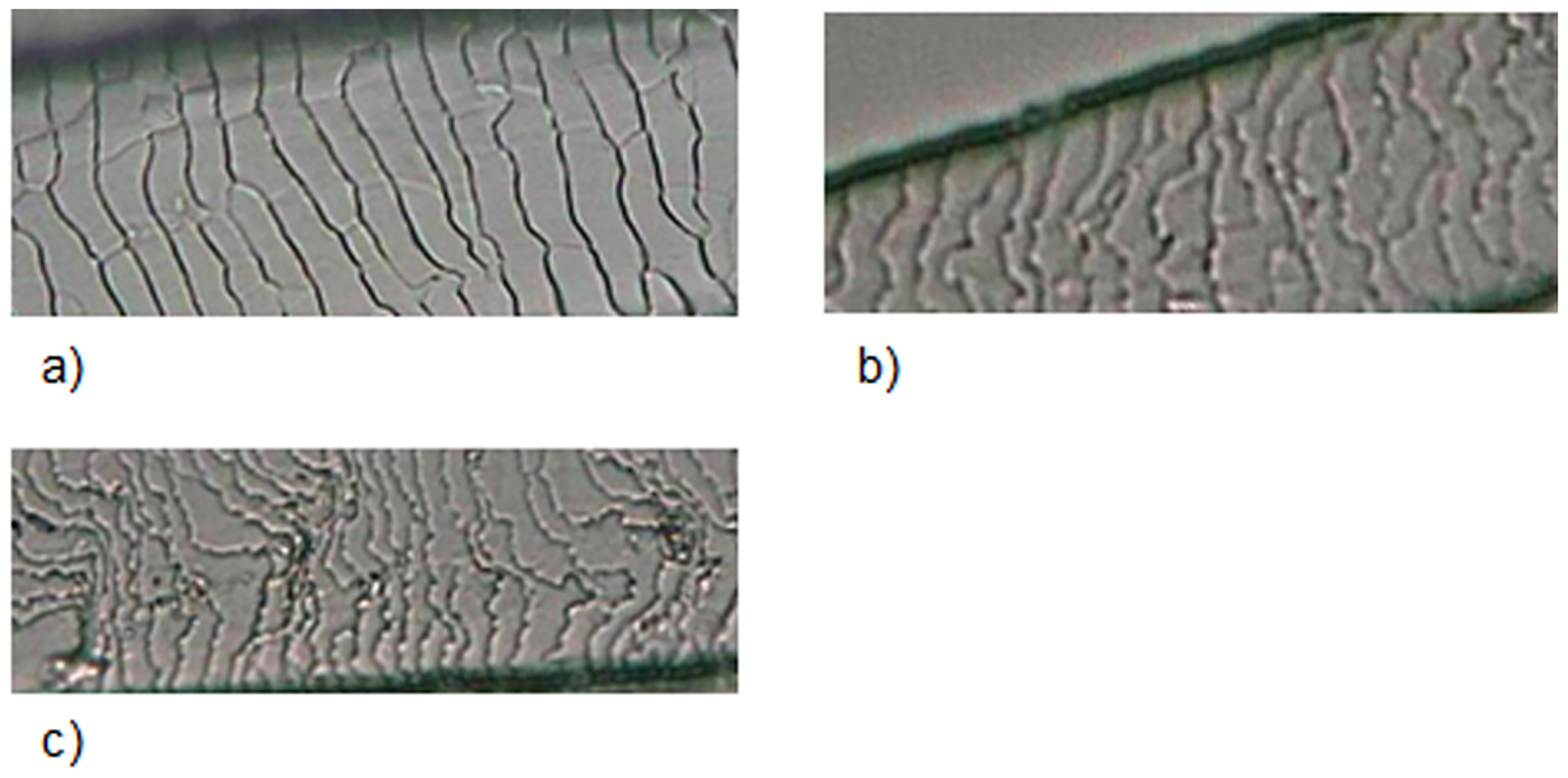
Cuticle scale patterns on the shaft of guard hairs of selected Neotropical felids. (a) regular wavy pattern; (b) irregular wavy pattern; (c) irregular wavy pattern with ornamented edges.

As in some previous studies [50–53], we also found that the shaft is the most important region for differentiating among species based on cuticle scales (Fig 3).

**Fig 4:**
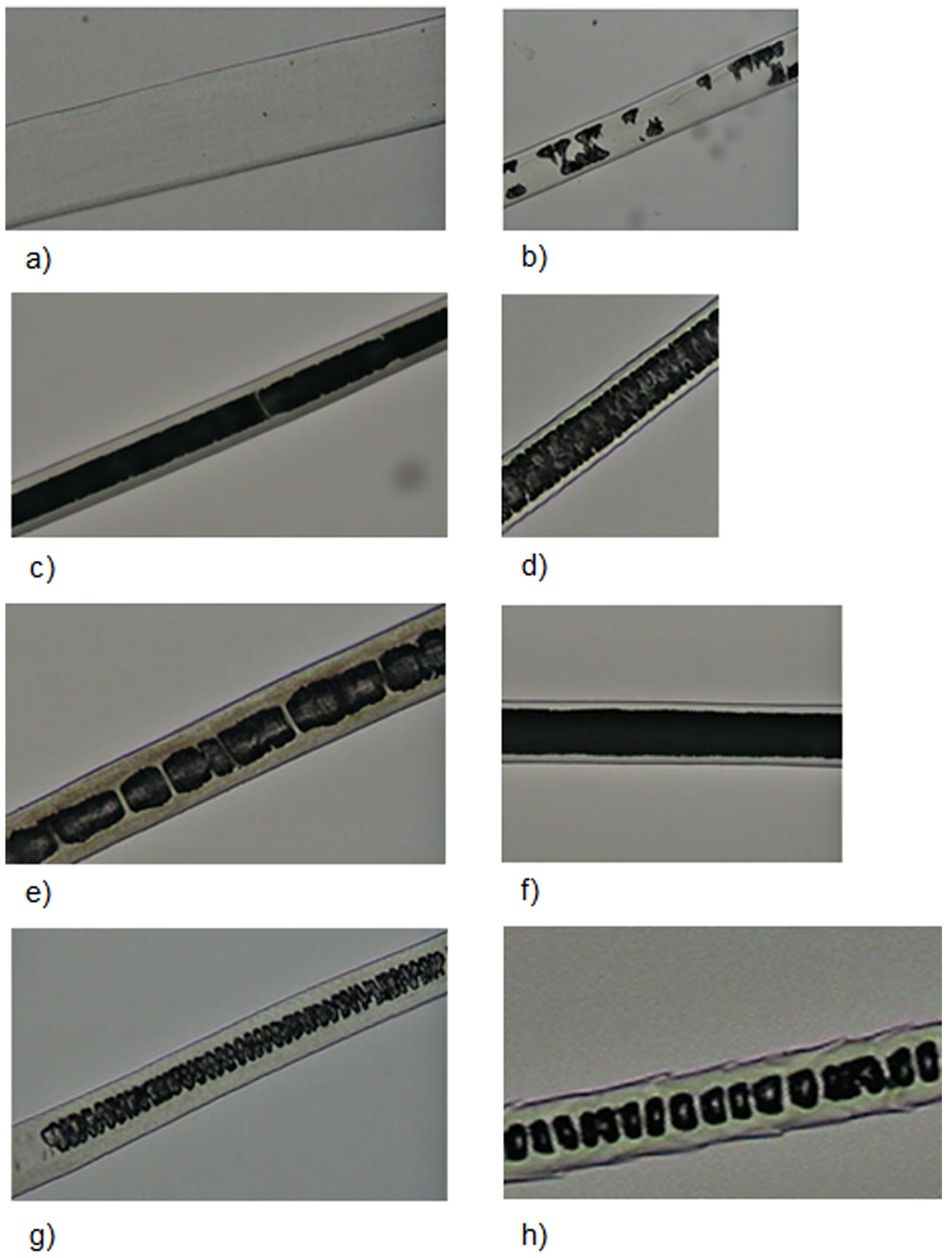
Medullar patterns on guard hairs of selected Neotropical felids. (a) absent; (b) fragmented; (c) continuous with filaments; (d) trabecular; (e) uniseriate ladder; (f) deeply pigmented continuous; (g) diagonal uniseriate; (h) small squares.

On the medulla (Fig 4), two regions were observed, shield and shaft. We considered three of their features: 1) Continuity: the medulla may be continuous or not; 2) Width: we compared the width of the medulla with that of the cortex; 3) Shape profile: diagonal uniseriate, with most cells arranged diagonally in relation to the hair axis, creating a morphological configuration similar to some alphabet letters, such as N, M, Y, H; trabecular, with the medulla showing a pattern of rodlike serial structures, parallel and adjacent to each other and transverse to the hair axis; uniseriate ladder, with cells forming regular or irregular squares or rectangles of variable size. In many cases, the medulla is nearly transparent, consisting mainly of air vacuoles, delimited by filaments.

Using these morphological patterns, we constructed the following hair identification key, which is an updated version from a previous monography [23]

### Identification key for guard hairs from eight Neotropical felids

**Fig 5:**
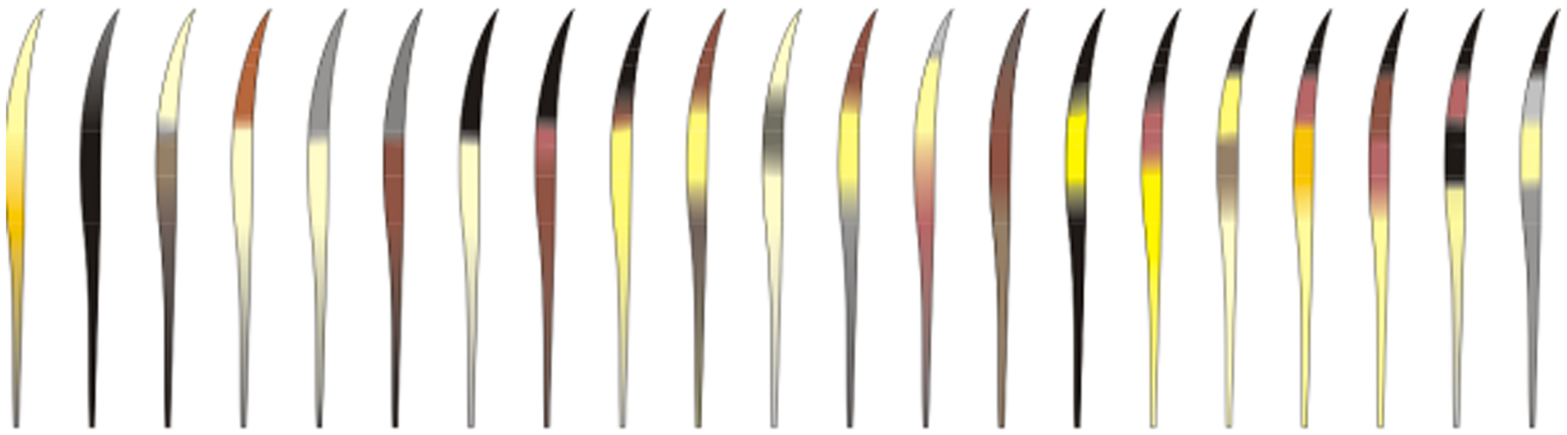
Guard hairs, color and banding patterns in *P*. *concolor*.

**Fig 6:**
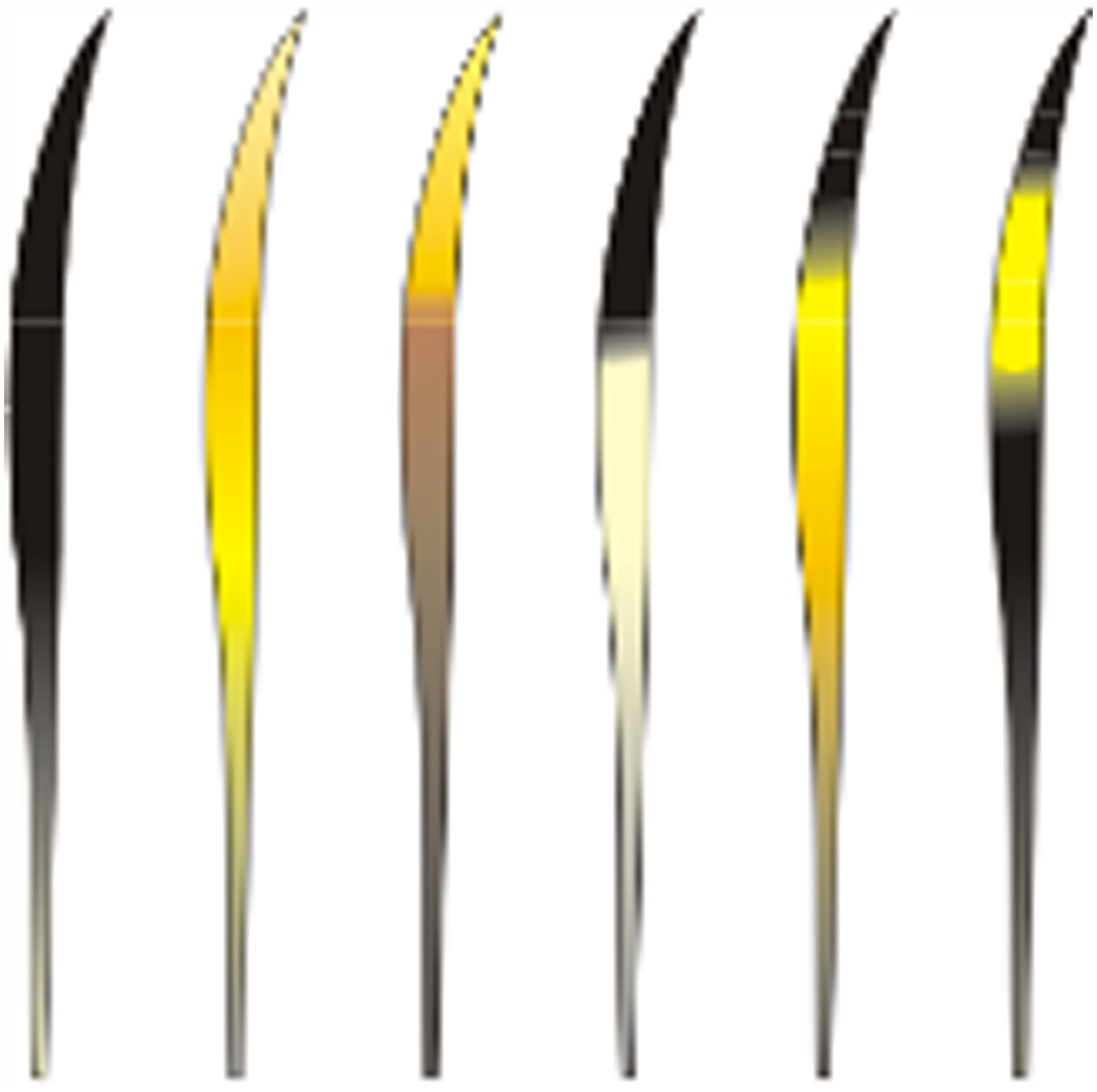
Guard hairs representation predominantly in yellow and black.

**Fig 7:**
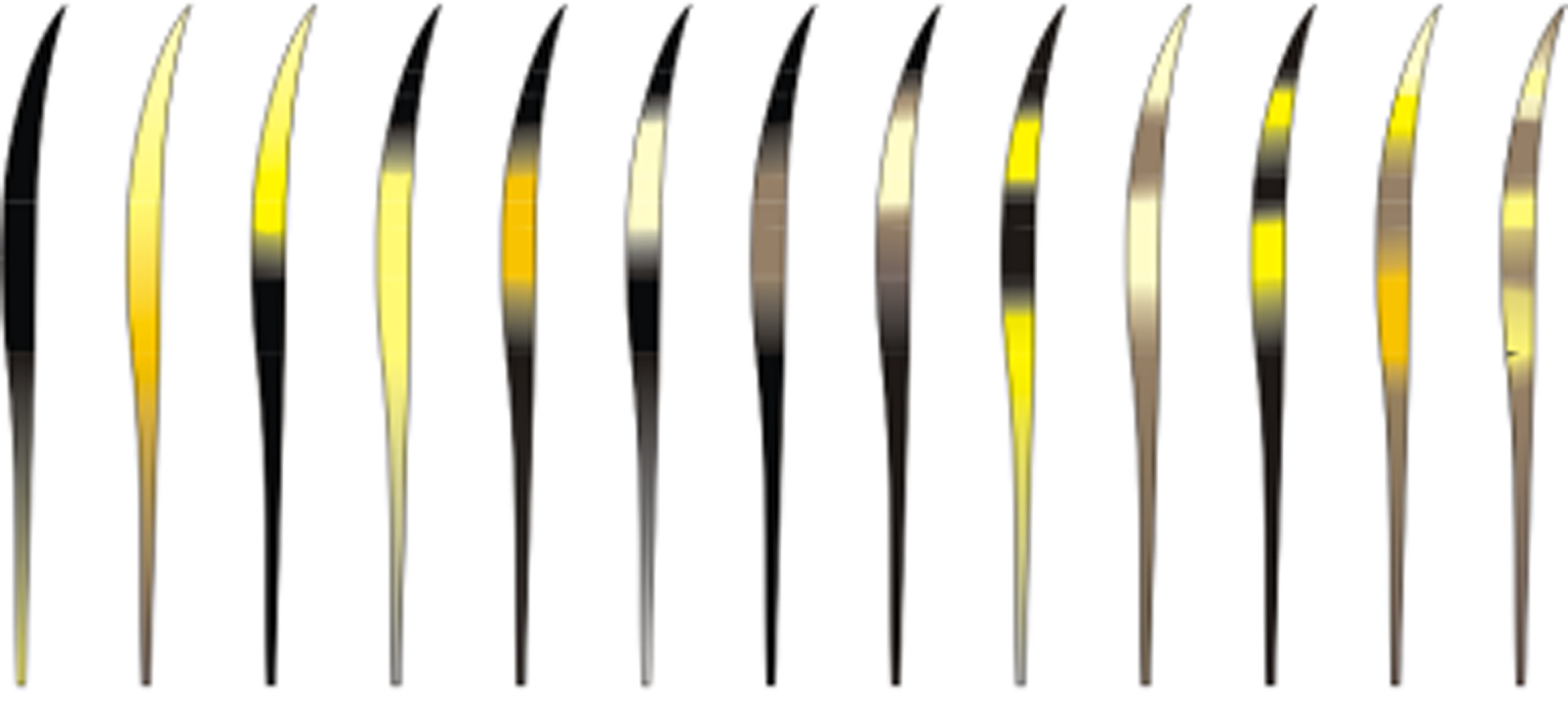
Guard hairs, color and banding patterns of *L*. *colocolo*.

**Fig 8:**
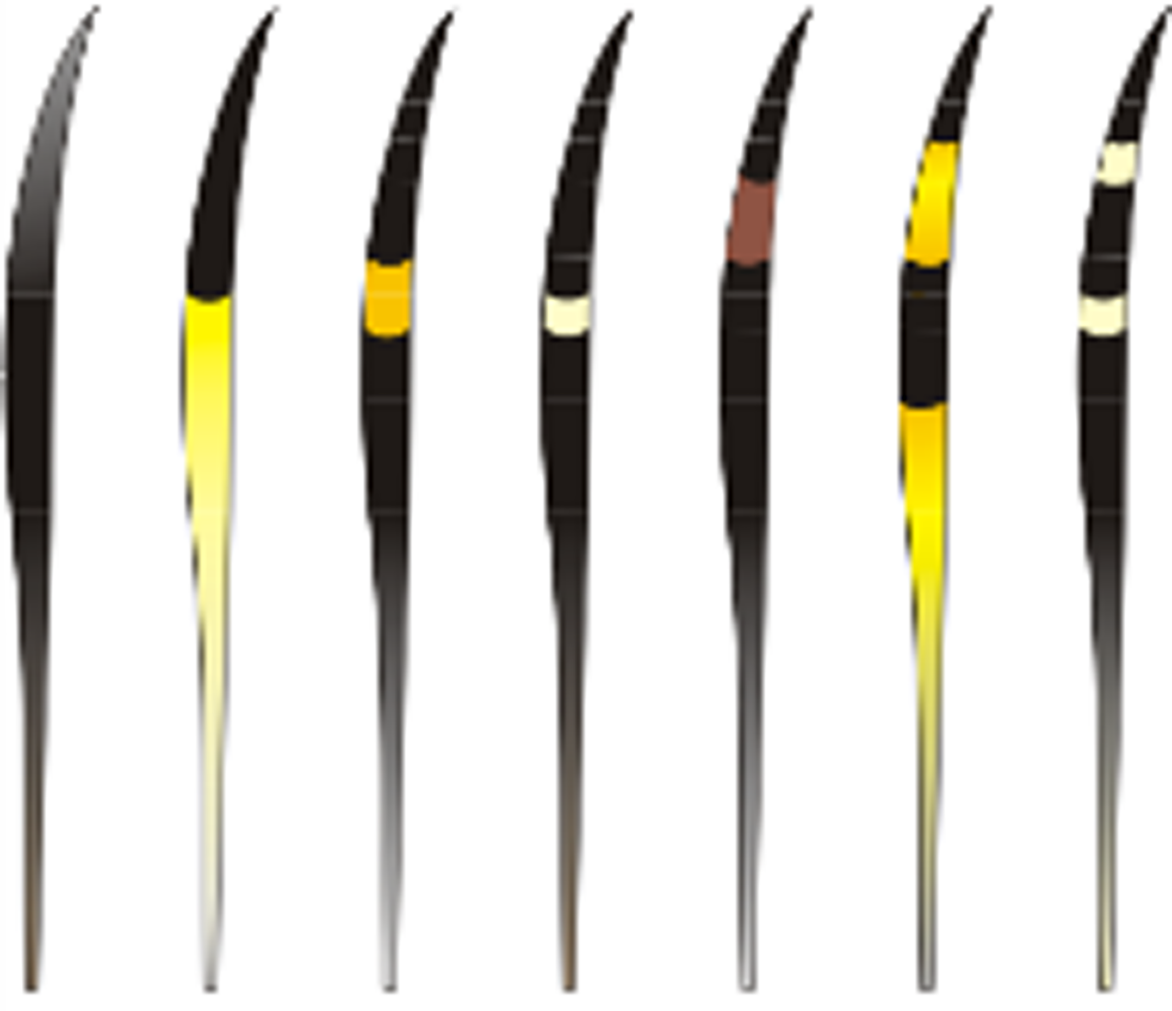
Guard hairs, color and banding patterns of *P*. *yagouaroundi*.

**Fig 9:**
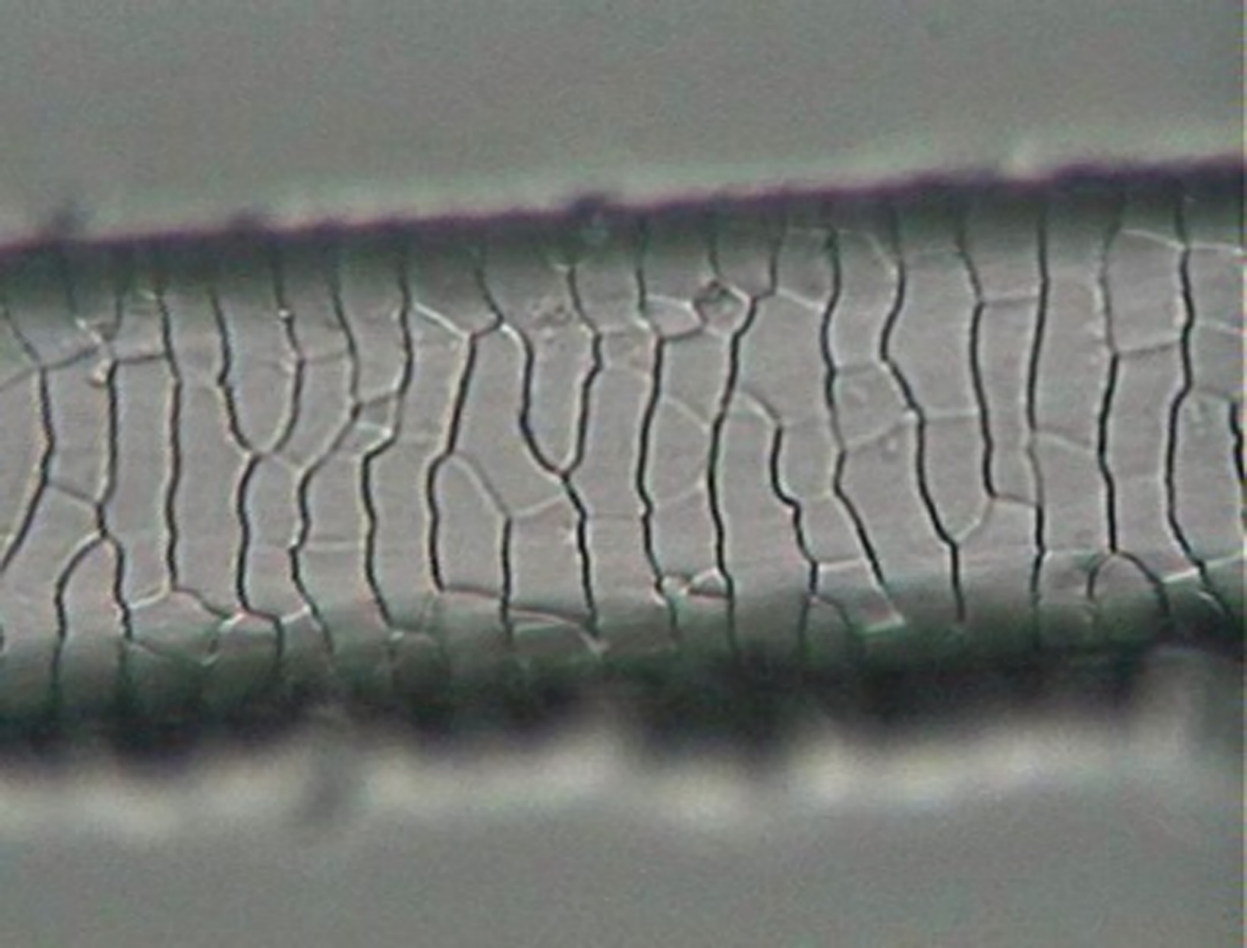
Scales, typical pattern in shield region of *P*. *onca*.

**Fig 10:**
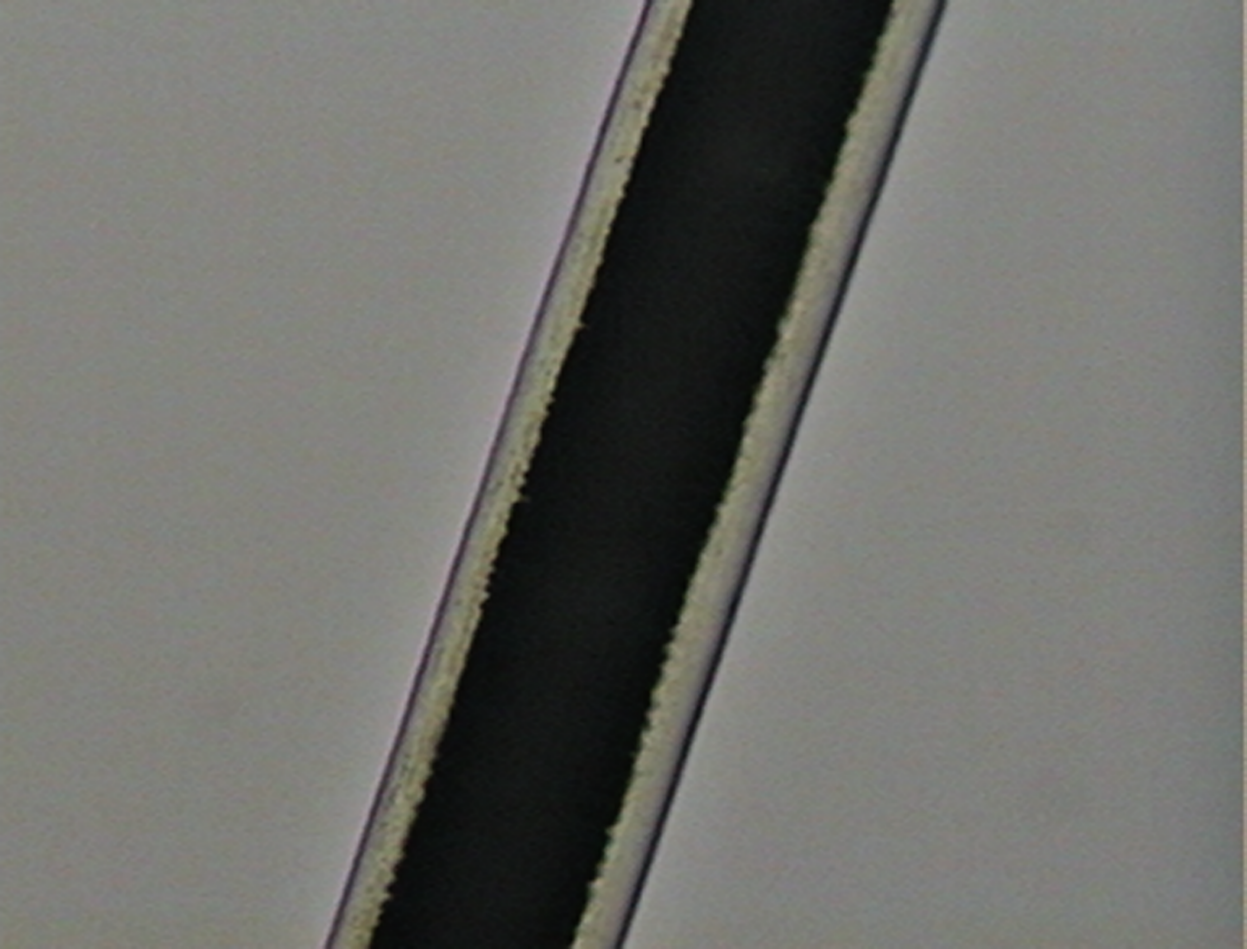
Medulla, typical pattern in shield region, in guard hairs of *L*. *tigrinus*.

**Fig 11:**
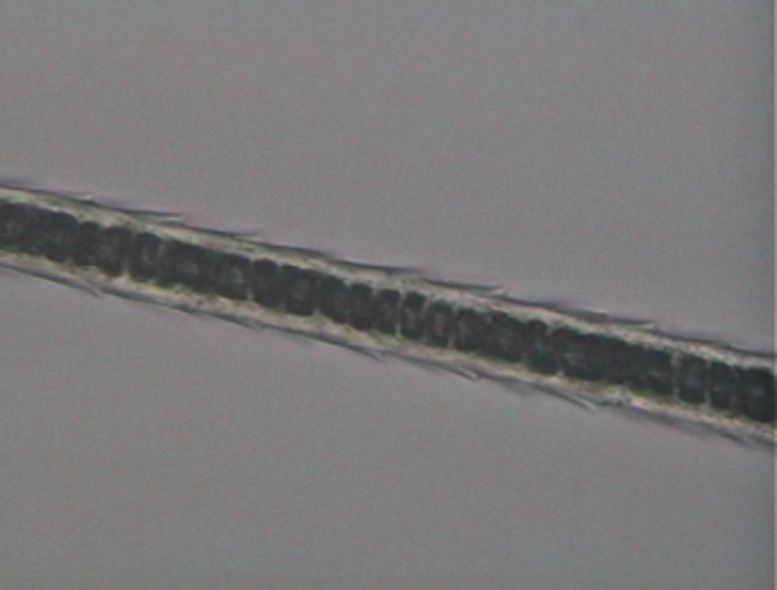
Medulla, typical pattern in shaft region from guard hairs of *L*. *tigrinus*.

**Fig 12:**
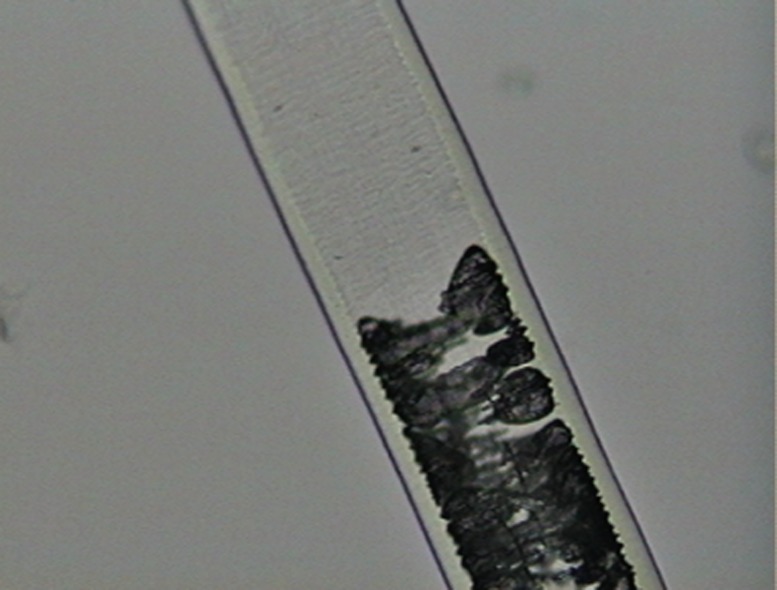
Medulla, typical pattern in shield region from guard hairs of *L*. *geoffroyi*.

**Fig 13:**
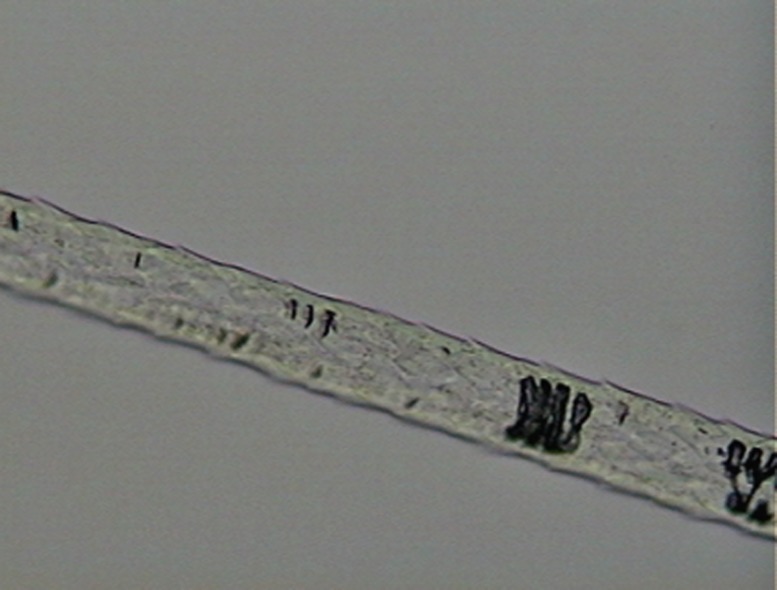
Medulla, typical pattern in shaft region from guard hairs of *L*. *geoffroyi*.

**Fig 14:**
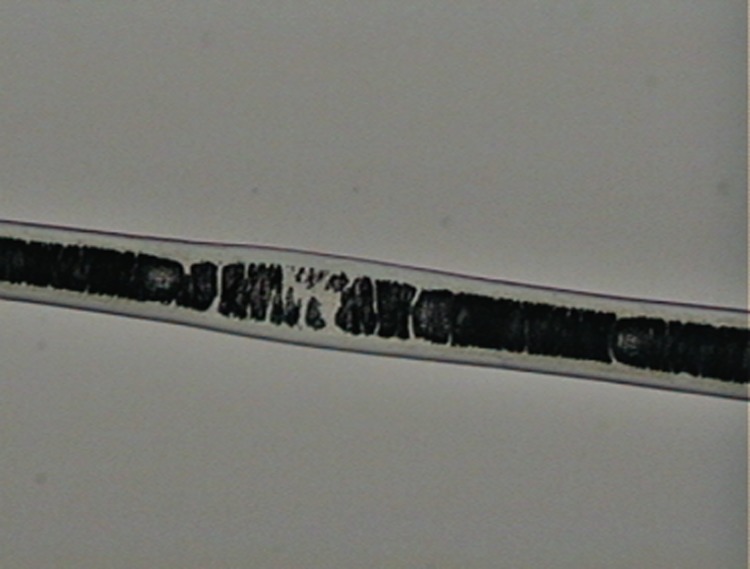
Medulla, typical pattern in shield region from guard hairs of *L*. *wiedii*.

**Fig 15:**
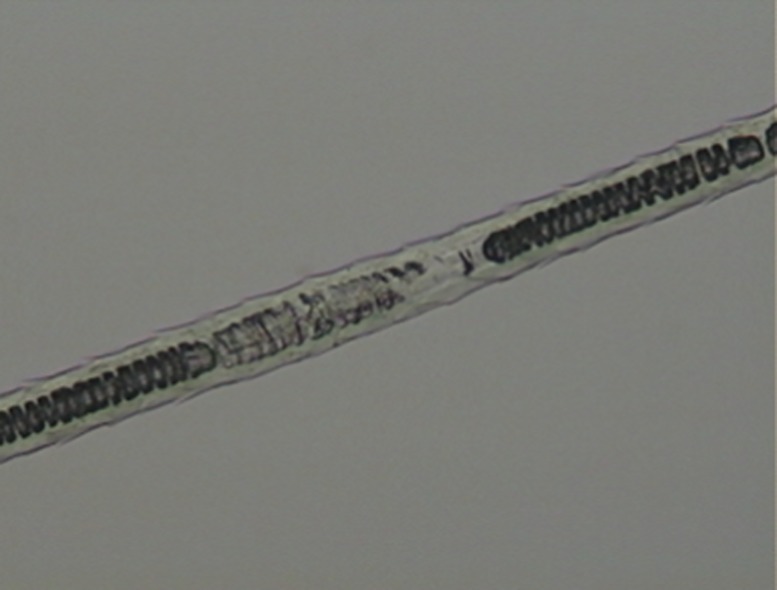
Medulla, typical pattern in shaft region from guard hairs of *L*. *wiedii*.

**Fig 16:**
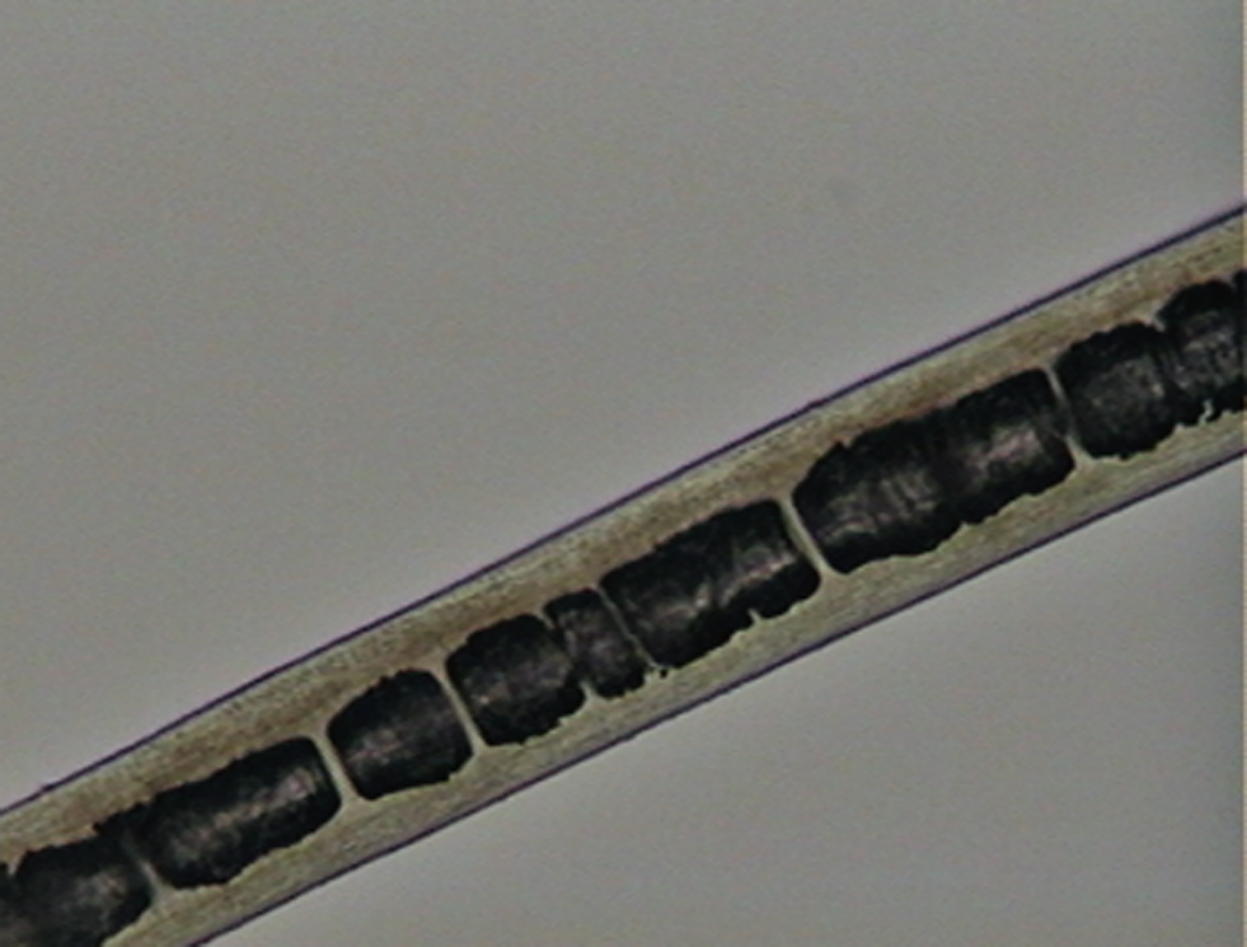
Medulla, typical pattern in shield region from guard hairs of *L*. *pardalis*.

**Fig 17:**
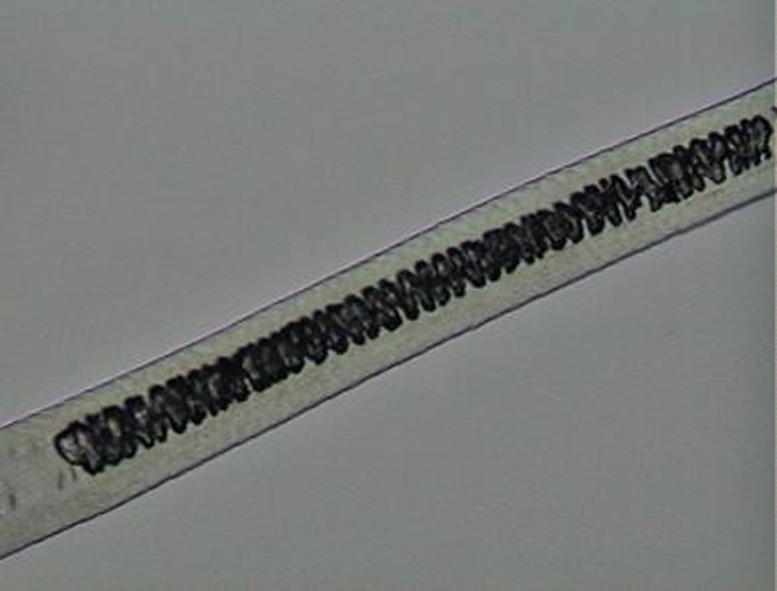
Medulla, typical pattern in shaft region from guard hairs of *L*. *pardalis*.

1)
  a) Hairs with maximum length about 2.0 cm to 4.0 cm, straight and thick, clearly distinguishable by their predominantly brownish tones (Fig 5)…***P*. *concolor***
  b) Hairs predominantly with yellow and black tones (Fig 6)…**2**
2)
  a) Hairs 4.5 cm or more in length and/or with five or more bands in the shield region, with alternate coloration in black (or dark brown) and yellow (Fig 7)…***L*. *colocolo***
  b) Hairs very thick, about 2.0 to 3.0 cm in diameter. With sharply defined, short, pale-colored (nearly white) bands in the shield region. May have up to 5 bands, more commonly 3 bands on the shield. with alternate colors of black and yellow (or cream-colored) (Fig 8)…***P*. *yagouaroundi***
  c) Hairs with at most four bands on the shield, variable in size, mainly black and yellow **OR** all hairs dark-colored (black and/or dark-brown)…**3**
3)
  a) Hairs with scales in the beginning of the shield region with regular wavy pattern and no ornamentation on the edge (Fig 9)…***P*. *onca***
  b) Hairs with scales in the beginning of the shield region with regular wavy pattern and moderate ornamentation on edge of scales **OR** same region with irregular wavy pattern with ornamented edges…**4**
4)
  a) Medulla in the shield region: width varying from two to five times as wide as the cortex, but generally three to four times as wide. Typically with a continuously and deeply pigmented medulla. Exceptionally there may be some hairs where the medulla is discontinuous or with long sections without medulla and/or with fragments, mostly in the beginning or end of the shield region (Fig 10).
  Medulla in the shaft region: Normally twice as wide as the cortex, but may vary from same width as cortex to up to four times as wide. Continuity: may vary from continuous to discontinuous and/or with long portions without medulla and/or with fragments and/or dotted. The form varies from diagonal uniseriate to the pattern of small squares (Fig 11)…***L*. *tigrinus***
  b) Medulla in the shield region: at least twice as wide as the cortex, generally three to six times as wide. In general, pattern discontinuous, with continuous portions—with trabecular or uniseriate ladder-shaped pattern—intercalated with long portions without medulla and/or long portions with fragments of medulla (Fig 12).
  Medulla in the shaft region: Normally three to four times as wide as the cortex, but with some hairs in which the medulla, in this region, is only slightly wider than the cortex. Normally discontinuous, with long portions without a medulla or with fragments of it; may have diagonal uniseriate or trabecular pattern, and have fragments with a dotted appearance. Rarely, some hairs may have a continuous medulla in this region (Fig 13)*…****L*. *geoffroyi***
  c) Medulla in the shield region: normally it is two to three times as wide as the cortex and continuous. More commonly its shape may be either of the trabecular pattern or the uniseriate ladder pattern. In some hairs, however, the shape may present both patterns, intercalated by each other. Sometimes, the uniseriate ladder pattern can also show a fragmented configuration with or without portions with no medulla. Rarely, a continuous and deeply pigmented medulla can be seen (Fig 14).
  Medulla in the shaft region: in this region, the medulla may be narrower than the cortex to up to twice as wide. Continuity may vary from continuous to discontinuous—with the diagonal uniseriate or trabecular pattern, or even patterned as a series of small squares, and/or with portions without a medulla and/or with fragments (Fig 15)…***L*. *wiedii***
  d) Medulla in the shield region: normally it is two to three times as wide as the cortex. In general it is continuous and presents the uniseriate ladder pattern, but there are some hairs in which the medulla shows sections with fragmented blocks and/or sections that lack a medulla (Fig 16).
  Medulla in the shaft region: in this region, the medulla may be narrower than the cortex to up to twice as wide. Continuity may vary from continuous—in the form of small squares—to discontinuous—with the diagonal uniseriate pattern—and/or with portions without a medulla and/or with fragments (Fig 17)…***L*. *pardalis***

### Testing the hair identification key

Table 3 shows the results obtained in the blind test of the Neotropical Felid Guard Hair Identification Key.

**Table 3:** Results of blind tests of hair identification key.

Species	No. samples	% success	Key effectiveness
*P*. *onca*	10	100	Excellent
*P*. *yagouaroundi*	10	100	Excellent
*P*. *concolor*	9	100	Excellent
*L*. *colocolo*	10	80	Excellent
*L*. *geoffroyi*	10	60	Good
*L*. *pardalis*	10	40	Moderate
*L*. *tigrinus*	10	40	Moderate
*L*.*wiedii*	10	30	Poor
**TOTAL**	**79**	**68.35**	**Good**

The effectiveness of the identification key can be considered excellent for detecting hairs of *P*. *onca*, *P*. *yagouaroundi*, *P*. *concolor* and *L*. *colocolo*, with success rates of 100%, 100%, 100% and 80% respectively. Indeed, the jaguarundi and puma can be easily recognized by the coloration and banding pattern of their guard hairs. The jaguar can also be effortlessly differentiated from the other species, since only this species has a continuous medulla, in the shape of blocks (or a uniseriate ladder, the alternate name for this pattern) in the shield portion of the guard hairs, with its characteristic width being less than that of the cortex. *L*. *colocolo* has most of its guard hairs much longer than those of the other species, about 45 mm. The results of the key test can be considered good, with 60% success, for *L*. *geoffroyi*. The results for the ocelot and oncilla can be considered moderate, with a success rate of 40%. Only for *L*. *wiedii* were the key results poor. The overall success rate of the key, for the group of specimens used here, was 68.35%.

Table 4 shows species with problems in the identification and the number of times and percentages with which they were confused with another species.

**Table 4:** Species confused with each other in the tests of the key.

Species	No. of times confused	% error
*L*. *tigrinus*, *L*. *geoffroyi*	6	35
*L*. *pardalis*, *L*. *wiedii*	4	20
*L*. *colocolo*, *L*. *wiedii*	2	15
*L*. *wiedii*, *L*. *geoffroyi*	3	15
*L*. *tigrinus*, *L*. *colocolo*	2	10
*L*. *tigrinus*, *L*. *pardalis*	2	10
*L*. *colocolo*, *L*. *geoffroyi*	1	5
*L*. *colocolo*, *L*. *pardalis*	1	5
*L*. *pardalis*, *L*. *geoffroyi*	1	5
*L*. *tigrinus*, *L*. *wiedii*	1	5

Table 4 lists the species with more-similar morphological features in their guard hair and which therefore can be confused with each other, leading to false-positive identifications. The final differentiation for *L*. *tigrinus*, *L*. *pardalis*, *L*. *wiedii* and *L*. *geoffroyi* can be made only at the medullar level. Errors with *L*. *colocolo* occurred only when guard hairs were less than 45 mm in length, and/or the hairs lacked a series of five bands in the shield region, in the following order: black, yellow, black, yellow. Both conditions occurred only rarely.

Although *L*. *tigrinus* and *L*. *geoffroyi* have distinct medullar patterns in most guard hairs, sometimes they share the same features, causing erroneous identifications. The same is true for the pair *L*. *pardalis* and *L*. *wiedii*, but to a greater extent. This is the reason for the relatively large number of misidentifications for the pair. Even though these two species were successfully discriminated three times from each other, they were misidentified in four other comparisons. Therefore, false-positive identifications are a real possibility, especially for *L*. *wiedii*, but can also occur with *L*. *geoffroyi* and *L*. *tigrinus*.

### Molecular marker test

Using specific primers to unravel the identity of species from fecal samples, we obtained 100% success for all eight species that were blind-tested (Table 5). The obtained sequences are available in S1 Table, in Supporting Information. There was no difference in the results for the test conditions of the samples, *i*.*e*. the results did not vary if the sample was preserved in ethanol and frozen immediately after it was collected (condition fresh/freeze), or if it was frozen only a week later (week/freeze), or if both preservation in ethanol and freezing were done seven days after the collection (week/week).

**Table 5:** Number of samples used for molecular blind-test. All tests were successful.

Species	No. samplesfresh/freeze	No. samplesweek/freeze	No. samplesweek/week	Total No. samples	% success	Efficiency
*P*. *onca*	3	3	3	9	100	Excellent
*P*. *yagouaroundi*	3	3	3	9	100	Excellent
*P*. *concolor*	3	3	3	9	100	Excellent
*L*. *colocolo*	3	3	3	9	100	Excellent
*L*. *geoffroyi*	3	3	3	9	100	Excellent
*L*. *pardalis*	3	3	3	9	100	Excellent
*L*. *tigrinus*	3	3	3	9	100	Excellent
*L*. *wiedii*	3	3	3	9	100	Excellent
**TOTAL**	**24**	**24**	**24**	**72**	**100**	**Excellent**

## Discussion

Our results from the blind test of the Neotropical felid hair identification key can be considered “good”, although with some limitations on its performance for certain species. However, previously published studies have reported greater limitations in identifying hairs than those of our method.

Our key yielded excellent results for *P*. *onca*, *P*. *concolor* and *P*. *yagouaroundi*. These species may have overlapping home ranges [54–56] and this information is important in many ways, but especially because in our approach the distribution may be a crucial way to avoid misidentifications.

In contrast to another report [57], our study did not find that the cuticle scale pattern was the most useful one to identify felids by their guard hairs. On the contrary, the color and banding patterns, followed by the medullar morphology were determinant for identification, whereas the scale patterns proved to be the least important aspect.

One report [20], on the other hand, seems to support our view of the importance of color (and banding) and medullar shape, since it compared only the scale patterns of the ocelot, margay, oncilla and jaguarundi, and was unable to distinguish them on this basis.

Our results also differ from those in another report [35], which could only distinguish the same species analyzed here as belonging to three general groups. In our view, these authors did not obtain equivalent results because they did not use the diagnostic characters provided by the color and banding patterns of guard hairs.

One investigator [58], instead, when studying Neotropical spotted felids, succeeded in discerning scats from *L*. *pardalis*, *L*. *wiedii* and *L*. *tigrinus*, using a method similar to ours. However, she stated that she also used the identification of footprints and scat morphology to aid identification.

As is apparent from our results, apart from the jaguar, puma and jaguarundi, which could be identified precisely with our key, and the margay, which could only occasionally be recognized, for four other species, *L*. *pardalis*, *L*. *geoffroyi*, *L*. *colocolo* and *L*. *tigrinus*, we obtained moderate or good results, using only the key. Auxiliary information about their known distributions, *i*.*e*. taking into account the location where the sample was found in the field, can improve the method substantially since the overlaps between the ranges of these species are small or nonexistent. Geoffroy’s cat occurs in the temperate Neotropics and along the Andes, while the Pampas cat shares part of this distribution but also occurs in central Brazil, in this case the savanna-like Cerrado biome, with both species almost never found in rainforest biomes such as the Amazon and Atlantic Forests. *L*. *tigrinus*, in its turn, inhabits these latter habitats, which produces an almost completely inverse distribution [22].

Using the key together with distribution criteria, it is difficult to differentiate only *L*. *wiedii* from *L*. *pardalis* and vice-versa, but there is no problem in discriminating both of them from all other species that share the same distribution. Published reports have neglected to consider the improvement that can be gained by using the geographical distribution together with the hair morphology for identification of these species. Using distribution information, we could not differentiate the ocelot from the margay, but we did manage to distinguish every other species. It has been suggested [59] that *L*. *wiedii* is a neotenic morph of *L*. *pardalis*, thus explaining the close similarities found in their general morphology, including their hairs.

Even though including geographical information can improve results where morphological misidentification is more likely, as we have shown here, one must be aware that the very nature of these animals, being secretive and difficult to study, on the one hand, and the great extent and diversity of biomes in the Neotropical Region, on the other, can lead to inaccurate information about their true distributions. Furthermore, human impacts can alter their former natural distributions in unnoticed ways. In summary, the strategy of using distribution data alongside hair morphology must be used with caution.

Other resources such as camera traps and sighting reports can help to resolve possible problems in identifying species with very similar hair morphology, as has been done by other authors [60] to differentiate ocelot scats from other possible felid species.

The blind tests of our key showed the limits of its efficacy, which allows prospective users to decide whether it is suited to a specific kind of fieldwork, since errors may well occur [19]. Advantages of our method are that it requires only a small amount of time and skill from the researcher; hair gathering, whether from scats or from other sources, can be accomplished alongside other field tasks in a survey; and samples can easily be stored and processed when convenient.

### Molecular identification

Through the use of our molecular method, we were able to identify all fecal samples regardless of the time that they remained in “field” conditions or how they had been processed, suggesting that our method avoided the DNA degradation issue, at least for the first week of age.

One study [60] estimated that degradation rates of DNA in pellet samples from American pronghorns (*Antilocapra americana*) increased over time when the pellets remained in the field. The success of primer amplification in samples collected and preserved on day 1 after they were deposited decreased from 81% to 63% at day 7, to 2% at day 14 and 0% at day 60. Another study [61] found that the success of primers for coyote (*Canis latrans*) nuclear DNA (nDNA) decreased to around 70% at day 21 when samples of scats were collected in winter, and to less than 50% at day 7 in the summer, in an area in the Nearctic Region. Still another report [62], studying three of the eight species analyzed here, found an nDNA PCR primer general amplification rate of 61%, and also that 49% of the samples were successfully identified for species and individual. Therefore, the results of our hair-morphology approach are equivalent to or better than those achieved by the molecular methods reported in recent publications, possibly due to DNA degradation when scat remains in field conditions for longer periods. That is to say, even though these are two distinct problems, the possibility of misidentification generating false positives by using hair morphology, and not amplifying the DNA from all samples, resulting in false negatives, the success rates for these two methods can be compared. PCR inhibitors present in fecal samples may constitute an obstacle for molecular analysis. DNA dilution can resolve this problem, as it reduces the concentration of the inhibitor in the PCR [63]. In this study, we used a DNA volume ranging from 3 μl to 5 μl to obtain 100% amplification success.

The results for our own molecular method indicated that, with this method, all species could be identified with 100% accuracy, with no false negatives, even if the samples were held under conditions similar to those in the field. Because we used a primer pair designed for annealing in conserved regions of the ATP6 gene [3] among individuals, we believe that the risk of misidentification is very low. The use of a reference sequence, as in our study, is also essential to avoid false positives or false negatives. In addition, we used only high-quality sequences (electropherogram without dubious peaks), which we consider vital for a reliable identification.

## Conclusion

Both methods assessed here gave better results than expected, even compared to other published results, since our trichology technique outperformed some molecular methods used in recent studies, and our own molecular approach performed even better.

These results increase confidence in both methods, which is essential when dealing with rare, elusive and endangered species, such as most of those analyzed here. This allows more freedom in the choice of approaches, depending on the objective of the study. If one seeks for more flexibility and lacks time and/or more elaborate laboratory resources, or needs a result when still in the field, one should choose trichology. If, on the other hand, one requires absolute accuracy and/or has the time and access to a genetics laboratory, then our molecular method is the appropriate option.

The most important new findings of this study are: 1) that trichology can indeed be reliably used to identify Neotropical felids, especially if distribution information is available and employed; and 2) our molecular procedure yields excellent results even if the samples are not fresh.

Besides increasing confidence in the outcome, independently of either method used, our results suggest a lower cost and a simpler procedure, in both the field and the laboratory, since greater accuracy means that fewer samples will be needed, which leads to less time spent collecting samples, fewer samples required to achieve results, and fewer resources needed to preserve, transport and analyze the samples.

## Supporting information

S1 Table Accession number (GenBank-NCBI) of the sequences obtained from the fecal samples for the ATP6 region. (PDF)
