# Correction: Comparing hair-morphology and molecular methods to identify fecal samples from Neotropical felids

**DOI:** 10.1371/journal.pone.0190758

**Published:** 2018-01-02

**Authors:** The PLOS ONE Staff

There are errors in the Funding section. The publisher apologizes for these errors. The correct funding information is as follows: The morphological aspects of this study were supported by two grants (Fapesp 04/08187-3 and Fapesp 98/10275-5) while the molecular features of it were supported by other two grants (Fapesp 2013/24453-4 and CNPq 308385/2014-4). IDEA WILD provided the license for the software GENEIOUS. The funders had no role in study design, data collection and analysis, decision to publish this work.
