# Supplementary material for: Comparing hair-morphology and molecular methods to identify fecal samples from Neotropical felids
Source: PLoS One. 2017 Sep 7;12(9):e0184073. doi: 10.1371/journal.pone.0184073 (PMC5589158; doi:10.1371/journal.pone.0184073)
Supplement: S1 Table — (PDF) [file pone.0184073.s001.pdf]

Table S1. Accession Number (GenBank-NCBI) of the sequences obtained from the fecal samples for the ATP6 region.

Fecal Sample	Species	Accession number-NCBI
Sample 1	*Puma concolor*	KY688433
Sample 2	*Leopardus pardalis*	MF175121
Sample 3	*Panthera onca*	KY688434
Sample 4	*Leopardus colocolo*	MF175125
Sample 5	*Leopardus wiedii*	MF175129
Sample 6	*Leopardus geoffroyi*	MF175133
Sample 7	*Leopardus tigrinus*	MF175137
Sample 8	*Leopardus tigrinus*	MF175138
Sample 9	*Leopardus tigrinus*	MF175139
Sample 10	*Leopardus colocolo*	MF175126
Sample 11	*Leopardus geoffroyi*	MF175134
Sample 12	*Leopardus pardalis*	MF175122
Sample 13	*Puma yagouaroundi*	KY688435
Sample 14	*Leopardus wiedii*	MF175130
Sample 15	*Leopardus wiedii*	MF175131
Sample 16	*Leopardus tigrinus*	MF175140
Sample 17	*Leopardus wiedii*	MF175132
Sample 18	*Puma yagouaroundi*	KY688436
Sample 19	*Puma yagouaroundi*	KY688437
Sample 20	*Puma concolor*	KY688438
Sample 21	*Puma concolor*	KY688439
Sample 22	*Puma concolor*	KY688440
Sample 23	*Leopardus pardalis*	MF175123
Sample 24	*Leopardus pardalis*	MF175124
Sample 25	*Panthera onca*	KY688441
Sample 26	*Panthera onca*	KY688442
Sample 27	*Panthera onca*	KY688443
Sample 28	*Puma yagouaroundi*	KY688444
Sample 29	*Leopardus colocolo*	MF175127
Sample 30	*Leopardus colocolo*	MF175128
Sample 31	*Leopardus geoffroyi*	MF175135
Sample 32	*Leopardus geoffroyi*	MF175136
